# Diagnostic and prognostic potential clustered miRNAs in bladder cancer

**DOI:** 10.1007/s13205-022-03225-z

**Published:** 2022-07-13

**Authors:** Akshay Pramod Ware, Shama Prasada Kabekkodu, Arun Chawla, Bobby Paul, Kapaettu Satyamoorthy

**Affiliations:** 1 Department of Bioinformatics, Manipal School of Life Sciences, Manipal Academy of Higher Education, Manipal, 576104 Karnataka India; 2 Department of Cell and Molecular Biology, Manipal School of Life Sciences, Manipal Academy of Higher Education, Manipal, 576104 Karnataka India; 3 Department of Urology, Kasturba Medical College, Manipal Academy of Higher Education, Manipal, 576104 Karnataka India

**Keywords:** Cancer informatics, CNV driven miRNAs, Cancer prognosis marker, Big data analysis, Automated pipeline

## Abstract

At specific genomic loci, miRNAs are in clusters and their association with copy number variations (CNVs) may exhibit abnormal expression in several cancers. Hence, the current study aims to understand the expression of miRNA clusters residing within CNVs and the regulation of their target genes in bladder cancer. To achieve this, we used extensive bioinformatics resources and performed an integrated analysis of recurrent CNVs, clustered miRNA expression, gene expression, and drug–gene interaction datasets. The study identified nine upregulated miRNA clusters that are residing on CNV gain regions and three miRNA clusters (hsa-mir-200c/mir-141, hsa-mir-216a/mir-217, and hsa-mir-15b/mir-16-2) are correlated with patient survival. These clustered miRNAs targeted 89 genes that were downregulated in bladder cancer. Moreover, network and gene enrichment analysis displayed 10 hub genes (CCND2, ETS1, FGF2, FN1, JAK2, JUN, KDR, NOTCH1, PTEN, and ZEB1) which have significant potential for diagnosis and prognosis of bladder cancer patients. Interestingly, hsa-mir-200c/mir-141 and hsa-mir-15b/mir-16-2 cluster candidates showed significant differences in their expression in stage-specific manner during cancer progression. Downregulation of NOTCH1 by hsa-mir-200c/mir-141 may also sensitize tumors to methotrexate thus suggesting potential chemotherapeutic options for bladder cancer subjects. To overcome some computational challenges and reduce the complexity in multistep big data analysis, we developed an automated pipeline called CmiRClustFinder v1.0 (https://github.com/msls-bioinfo/CmiRClustFinder_v1.0), which can perform integrated data analysis of 35 TCGA cancer types.

**Supplementary Information:**

The online version contains supplementary material available at 10.1007/s13205-022-03225-z.

## Introduction

Carcinoma in the epithelial lining of the urinary bladder has been increasing over the years (Sarkis et al. 2020). Globally, 573,278 bladder cancer (BCa) cases and associated 212,536 deaths were reported in 2020 (Sung et al. 2021). Traditional techniques for the diagnosis, prognosis, and monitoring of BCa include imaging tests (ultrasound, computerized tomography (CT), magnetic resonance imaging (MRI)), cystoscopy, and urine cytology. However, these methods fall short of expectations due to high cost, poor cytology sensitivity, high invasiveness of cystoscopy, and significant inter and intra-observer variability in tumor stage and grade interpretation (Su et al. 2019). The food and drug administration (FDA) has approved urinary biomarkers include BTA Stat, BTA Trak, nuclear matrix protein 22 (NMP22), UroVysion, ubiquitin C (UBC), and other assays such as immunocytochemistry (uCyt+ and DD23) and fluorescence in situ hybridization (FISH) being widely used for patient follow-up, though with lesser potency in low-grade cancer detection (Charpentier et al. 2021). BCa is generally managed with classical approaches such as chemotherapy, surgery, or radiation (Dobruch and Oszczudłowski 2021). Genetic and epigenetic alterations such as aberrant DNA methylation, altered chromatin remodeling, and dysregulated non-coding RNAs drive several molecular events during pathogenesis thereby contributing to varied clinical behavior in cancer recurrence and progression (Li et al. 2016; Martinez et al. 2019). Collectively, identifying novel biomarkers for prognosis and therapy is crucial to improving BCa patient care.

MicroRNAs (miRNAs) are approximately 20–23nt long, highly conserved, and non-coding RNAs that play an important role in the regulation of gene expression. The miRBase (v22.1) currently consists of 2654 mature miRNAs coded from 1917 precursor miRNAs. Recent studies have shown that the human genome contains 159 miRNA clusters that comprise 468 miRNAs (Kozomara et al. 2019). A miRNA cluster consists of two or more miRNAs located in physically adjacent regions and transcribed in the same orientation. Differentially expressed clustered miRNAs have been reported. For example, cluster hsa-mir-143/mir-145 on chromosome location 5q32 is downregulated in several cancers (Das and Pillai 2015). Clinical and mechanistic studies have shown that the deregulated clustered miRNA expression may play a crucial role in the pathogenesis of BCa (Braicu et al. 2019). Therefore, differentially expressed clustered miRNAs can be used for diagnostic and prognostic purposes in BCa.

Recent studies have reported that copy number variations (CNVs) are associated with aneuploidies and chromothripsis (Ben-David and Amon 2020) and are being recognized as an important risk factor for cancer, as they can alter the expression of their resident coding and non-coding genes (Shao et al. 2019). The miRNAs which reside on the CNV loci show a significant difference in expression patterns, especially in cancer conditions (Anauate et al. 2019). More than 50% of the miRNA genes are reported to overlap with the cancer-hotspot genomic regions and form a central regulatory unit in cancer development pathways (Farazi et al. 2013). The systematic array-based study by An et al. (2013) reported upregulation of two miRNA clusters (hsa-mir-23a/mir-24-2 and hsa-mir-181c/mir-181d) at chromosomal region 19p13.13 due to CNV amplification in gastric cancer. Overexpression of the largest human miRNA cluster ‘C19MC’ has been linked to a variety of cancers, including breast cancer (Jinesh et al. 2018), brain tumors (Sin-Chan et al. 2019) and thyroid adenomas (Rippe et al. 2010). The co-localization of moderately explored CNVs and miRNAs has indicated the potential of CNV-mediated variation in C19MC miRNA dosage (Vaira et al. 2012).

However, there are no comprehensive studies on CNV regulated clustered miRNA expression and its contribution to BCa. Hence, we performed an integrated analysis to identify potential clustered miRNAs to screen BCa. To meet the objectives, we performed an integrated analysis using a) clustered miRNAs residing on CNVs, b) patient survival, c) miRNA targeted genes, d) gene function and e) drug–gene interaction. We have developed a user-friendly computational pipeline named ‘CmiRClustFinder v1.0’ by integrating R and shell scripts. The pipeline can be effectively used for high throughput data analytics and to identify biomarkers for cancer diagnosis.

## Materials and methods

### Acquisition of miRNA expression and CNV data from bladder carcinoma

Level 3 miRNA expression datasets of BCa were interrogated from the TCGA database (https://portal.gdc.cancer.gov/projects/TCGA-BLCA). We analyzed 412 BCa patients data, of which 304 are males and 108 are females. The data belong to White (327 samples), Asian (44), Black or African-American (23) populations, and 18 samples population information was not available. Pre-computed Somatic copy-number alterations (SCNA) data without germline copy number amplification (CNA) was obtained from the Broad Institute’s FireBrowse portal (http://firebrowse.org/). Further, 468 miRNAs belonging to 159 clusters were retrieved from the miRBase V22.1 (http://www.mirbase.org/) database.

### Identification of SCNA and miRNA cluster co-occurrence

Recurrent SCNAs in the TCGA-BLCA samples were analyzed using Bioconductor package GAIA 3.10 (https://bioconductor.org/packages/release/bioc/html/gaia.html). Recurrent CNV was defined by false discovery rate (FDR) Q < 0.15 derived from 10 iterations. The segment mean of 0.3 was set as the threshold to identify the copy number gain/loss. The regions with segment mean > 0.3 and ≤ 0.3 thresholds were considered as a copy number gain (amplification) and loss (deletion) respectively. Finally, the genomic SCNA plot was generated using an R script with the cut-off value < 0.15 FDR. The GAIA uses human genome assembly Hg19 for all the analyses. Hence, we used the UCSC LiftOver (https://genome.ucsc.edu/cgi-bin/hgLiftOver) option to lift all SCNA genomic coordinates to match with the Hg38 build. Further, the functionality of BEDTools (Quinlan and Hall 2010) was used to intersect the genomic coordinates of miRNA clusters onto the recurrent significant CNV regions identified from GAIA analysis.

### The miRNA differential expression analysis

We used various functions of the ‘TCGAanalyze_DEA’ and TCGAbiolinks packages (Robinson et al. 2010) to identify differentially expressed miRNAs (Normal vs. Tumor). Further, using the false discovery rate (FDR) correction, the p value was adjusted to shortlist the top differentially expressed miRNAs. The logFC > 1 and FDR < 0.05 threshold were considered to be significant.

### Identification of clustered miRNA expression residing on CNV

Following criteria were used to identify the differentially expressed miRNA clusters residing on CNV regions: (i) at least two miRNAs must be differentially expressed from a cluster, and (ii) differentially expressed miRNA clusters must reside on copy number amplified or deleted regions. Inversely correlated cluster expression with CNVs and clusters with non-significant expression were excluded from the further analysis.

### Identification of prognostic signatures

The BCa samples were classified into low and high expression groups to identify the prognostically significant miRNA clusters according to the median miRNA expression levels. The Kaplan–Meier plotter hosted by the miRpower tool (Lánczky et al. 2016) was used to determine the relapse-free survival (RFS) with different clinical parameters. For miRNA cluster expression analysis, an unpaired *t* test was performed using MedCalc version 15.0 (Schoonjans et al. 1995). The *p* value < 0.05 was considered statistically significant. The plotted curves such as receiving operator characteristic (ROC) and areas under ROC (AUROC) were evaluated by comparing the values from tumor and normal tissues.

### miRNA target prediction and regulatory network analysis

The target genes of survival correlated miRNAs were obtained from miRTarBase (Huang et al. 2020), TargetScan (Garcia et al. 2011), DIANA-TarBase (Vlachos et al. 2015), mirDIP (Tokar et al. 2018), and miRDB (Chen and Wang 2020). Target genes commonly found in these five databases were only considered for further downstream analysis. The target gene expression and gene promoter methylation information were mined from the UALCAN database (http://ualcan.path.uab.edu/). Networks of downregulated genes targeted by clustered miRNAs were plotted. Further, these genes were examined for DNA promoter methylation to confirm the miRNAs target effect on gene expression. Using the beta value given in the UALCAN web server, DNA methylation was estimated in a range of 0 (unmethylated) to 1 (methylated).

### Protein–protein interaction (PPI) network analysis

The clustered miRNAs and their target genes network interaction were analyzed with GeneMANIA (https://genemania.org/) to explore the co-expression, co-localization, and shared protein domain information. The PPI networks were constructed using STRING V11.0 (Szklarczyk et al. 2017) with a medium confidence score ≥ 0.40 to predict the most interactive genes. The PPI network was imported and visualized using the Cytoscape plugin StringApp (Doncheva et al. 2018). Using CytoHubba (Chin et al. 2014), the top 10 hub genes were identified based on the distribution of network node degrees.

### Gene set enrichment analysis

We performed the functional enrichment analysis for 89 target genes using DAVID (https://david.ncifcrf.gov/) platform. The *p* value calculated using the Benjamini–Hochberg method and ≤ 0.05 threshold was considered statistically significant.

### Tissue, molecular-subtype, and clinical traits specific expression analysis

The present study used bladder cancer (404 samples) and normal tissue (28 samples) datasets available at the GEPIA server (Tang et al. 2017). The hub genes differentially expressed with a *p* value < 0.01 were considered statistically significant. The boxplot was used to illustrate the association between cancer and normal tissues. We used 412 BCa patient data available at the TCGA-GDC portal for the molecular subtype-specific expression analysis. These datasets comprised into five subtypes, basal-squamous (*n* = 142), luminal-papillary (*n* = 142), luminal-infiltrated (*n* = 78), luminal (*n* = 26), neuronal (*n* = 20) and four samples were uncategorized. To gain better insights, we have studied the BCa subtype-specific expression profile for three miRNA clusters and 10 hub genes. Tobacco smoking is a high-risk factor for carcinogenesis, and it can affect various organs such as the head, neck, lungs, and urinary bladder. Also, smoking induces the expression of various miRNAs, which post-transcriptionally silence the function of tumor suppressors and promote cancer (Fujii et al. 2018). Altered miRNA expression contributes to tumor growth and plays a critical role in response to chemotherapy (Li et al. 2013). Hence, we have studied the clustered miRNA and hub gene expression profiles in patients categorized into (i) smokers and non-smokers, (ii) chemotherapy responders, and non-responders.

### In silico validation of miRNA and gene expression

To validate the expression of clustered miRNAs and their target genes, we have used one miRNA—GSE36121 (Ratert et al. 2012); and three mRNA datasets—GSE40355 (Hecker et al. 2013), GSE27448 (Lambrou et al. 2013), GSE52519 (Borisov et al. 2018). These datasets were considered as the comprehensive reference for the miRNA and gene expression studies in urothelial carcinomas (Normal vs Tumor). The R package Limma-based online program GEO2R (https://www.ncbi.nlm.nih.gov/geo/geo2r/) was used to perform differential expression. The *p* value < 0.05 and logFC > 1 considered as significant.

### Hub-genes and drug interaction

The hub genes were analyzed with the DGIdb database (http://www.dgidb.org/) to understand the interrelation between drug candidates and genes. The analysis was restricted to the drugs approved by the Food and Drug Administration (FDA). Further, the PanDrugs (https://www.pandrugs.org/) database was used to identify potentially druggable molecular alterations and prioritization of anticancer drugs.

## Results

### Identification of CNV driven miRNA clusters

**Fig. 1:**
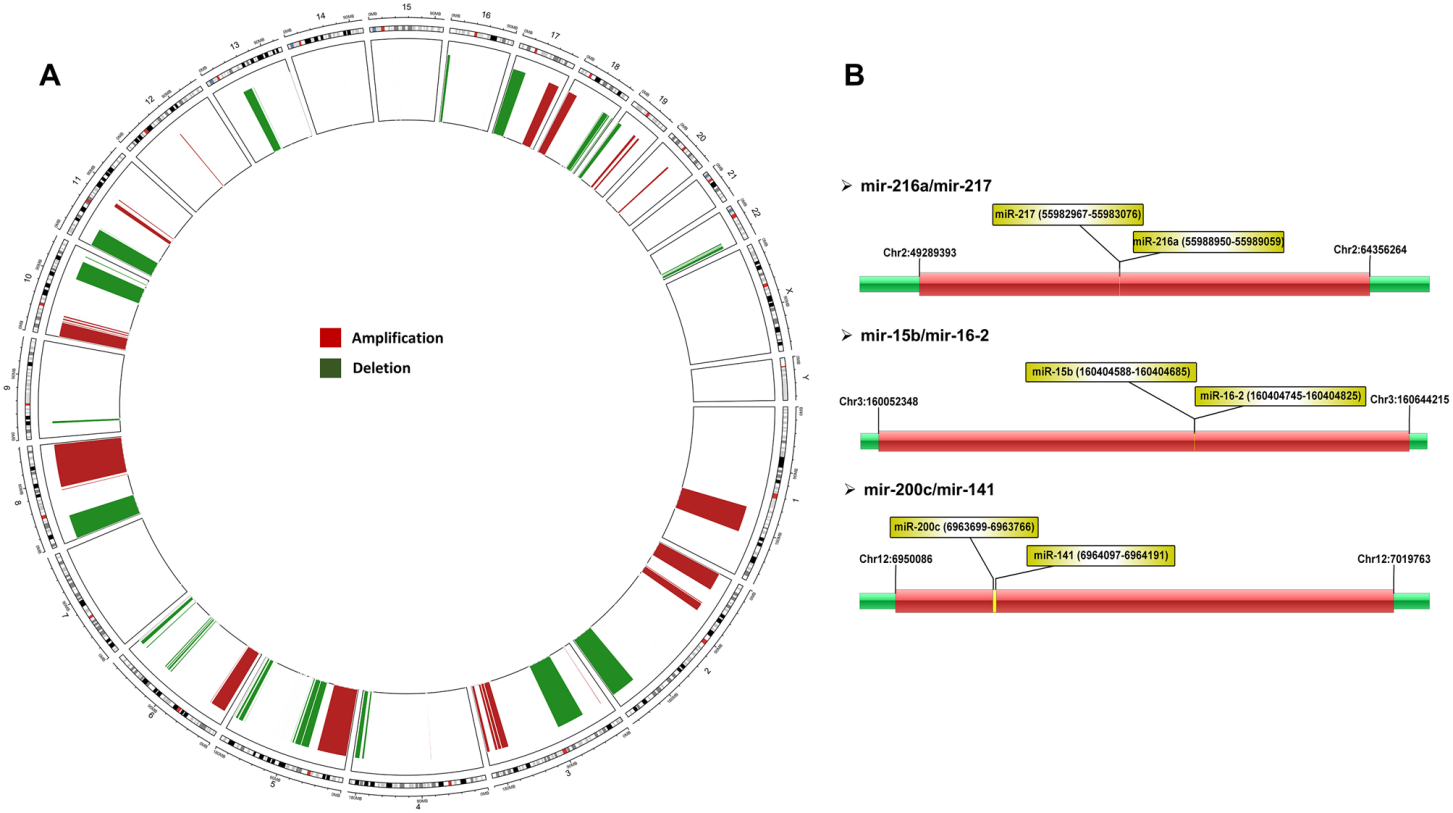
The distribution of recurrent copy number amplification and deletion in BCa. **a** The circos representation of CNV regions residing on human chromosomes (red: amplification; green: deletion). **b** Representation of three miRNA clusters (hsa-mir-200c/141, hsa-mir-216a/mir-217 and hsa-mir-15b/mir-16-2) residing on CNV amplified regions

The GAIA analysis of BCa-SCNA data resulted in 4119 significant CNV aberrations, of which 1824 regions were amplified, and 2295 regions were deleted (Supplementary Table S1, Fig. 1A). The mapping of 159 miRNA clusters on CNV regions showed overlapping of 61 clusters with CNV regions (Supplementary Table S2). Of the 61 miRNA clusters residing on CNV regions, 33 and 28 were located on amplified and deleted regions, respectively. Further, the expression analysis of quantile normalized BCa vs normal samples displayed 661 upregulated and 33 downregulated miRNAs. Using an integrated analysis, we identified 41 miRNAs belongs to nine upregulated miRNA clusters (hsa-mir-7113/mir-4691, hsa-mir-200c/mir-141, hsa-mir-3913-1/mir-3913-2, hsa-mir-657/mir-1250, hsa-mir-512-1/mir-526a-1, hsa-mir-371a/mir-373, hsa-mir-6804/mir-6803, hsa-mir-217/mir-216a and hsa-mir-15b/mir-16-2) which reside on the CNV gain regions (Supplementary Tables S3 & S4). However, no statistically significant expression was observed for miRNA residing on CNV deleted regions.

### Identification of the prognostic signature

**Fig. 2:**
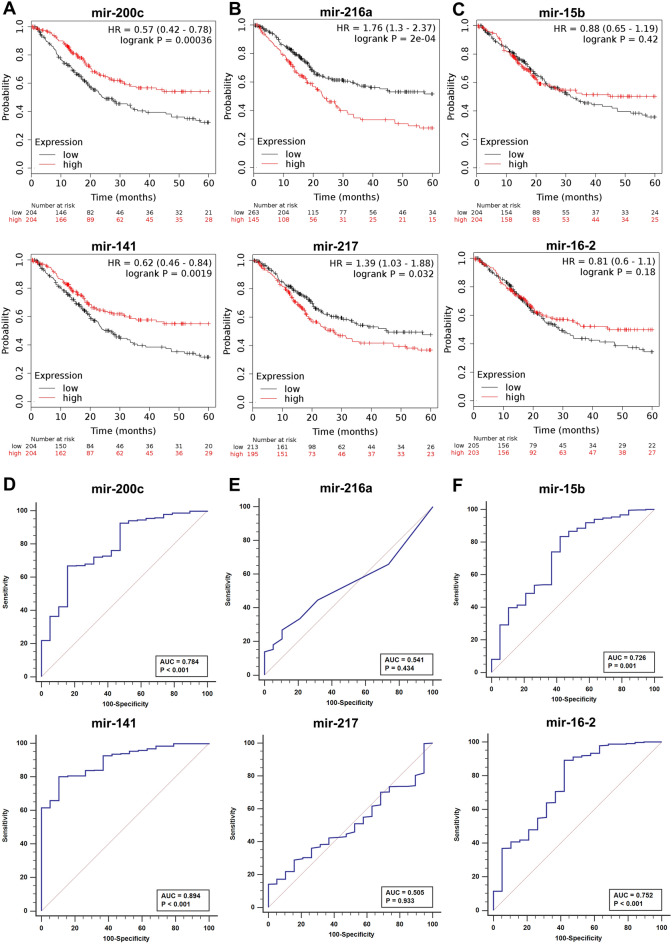
Prognostic feature analysis of three miRNA cluster candidates in BCa patients. The Kaplan–Meier survival curves show overall survival outcomes of miRNA cluster candidates. **a** mir-200c/mir-141, **b** mir-216a/mir-217, **c** mir-15b/mir-16-2 according to their expression in high and low-risk patient groups (black: low expression; red: high expression). The ROC curve showing diagnostic values of miRNA cluster candidates, **d** mir-200c/mir-141, **e** mir-15b/mir-16-2, and **f** mir-216a/mir-217. The ROC and AUROC curves are generated through MedCalc software

The Kaplan–Meier survival analysis was performed to identify the impact of CNV driven miRNA clusters on survival rate of BCa patients. We have compared survival analysis and hazard ratio with populations designated as miRNA clusters high and low risk in the TCGA database. The combined KM and ROC curve analysis identified three prognostically important miRNA clusters hsa-mir-200c/mir-141, hsa-mir-216a/mir-217, and hsa-mir-15b/mir-16-2 (Fig. 1B). The three miRNA clusters consist of six miRNAs were found to be upregulated (Supplementary Table S4). The overall survival rate of candidates of the hsa-mir-200c/mir-141 cluster is—hsa-mir-200c: HR = 0.57, CI = 0.42–0.78, *p* = 0.00036; hsa-mir-141: HR = 0.62, CI = 0.46–0.084, *p* = 0.0019. The candidate miRNAs of cluster hsa-mir-216a/mir-217 is—hsa-mir-216a: HR = 1.76, CI = 1.3–2.37, *p* = 2e-04; hsa-mir-217: HR = 1.39, CI = 1.03–1.88, *p* = 0.032 and for cluster hsa-mir-15b/mir-16-2 is—hsa-mir-15b: HR = 0.88, CI = 0.65–1.19, *p* = 0.42; hsa-mir-16-2: HR = 0.81, CI = 0.6–1.1, *p* = 0.18. The hsa-mir-200c/mir-141 and hsa-mir-216a/mir-217 (hsa-mir-200c: 2.01 LogFC; hsa-miR-141: 3.09 LogFC; hsa-mir-216a: 2.69 LogFC; hsa-mir-217: 2.45 LogFC) showed a significant correlation in the BCa patient survival (Fig. 2A, B). On the other hand, the hsa-mir-15b/mir-16-2 (hsa-mir-15b: 1.49 LogFC, hsa-mir-16-2: 1.52 LogFC) was not statistically significant for the survival of BCa patients (Fig. 2C). The higher expression of the hsa-mir-200c/mir-141 correlated with a better survival rate. However, hsa-mir-216a/mir-217 overexpression lead to a significant reduction in patient’s survival. We evaluated diagnostic value of CNV clusters by ROC curve analysis. The results indicated that the candidates of miRNA clusters hsa-mir-200c/mir-141 and hsa-mir-15b/mir-16-2 exhibited high diagnostic value (Fig. 2D, E). The area under the ROC curve (AUROC) for candidate miRNAs of these clusters were 0.784 (hsa-mir-200c), 0.894 (hsa-mir-141), 0.726 (hsa-mir-15b) and 0.752 (hsa-mir-16-2). The hsa-mir-216a/mir-217 cluster candidates harbored lower specificity and sensitivity (Fig. 2F) than the other two clusters. The conclusive KM and ROC results indicated that the prognostic model of members of these three clusters was robust in predicting the progression of cancer cell growth in BCa patients.

### Prediction of miRNA target genes and construction of regulatory networks

**Fig. 3:**
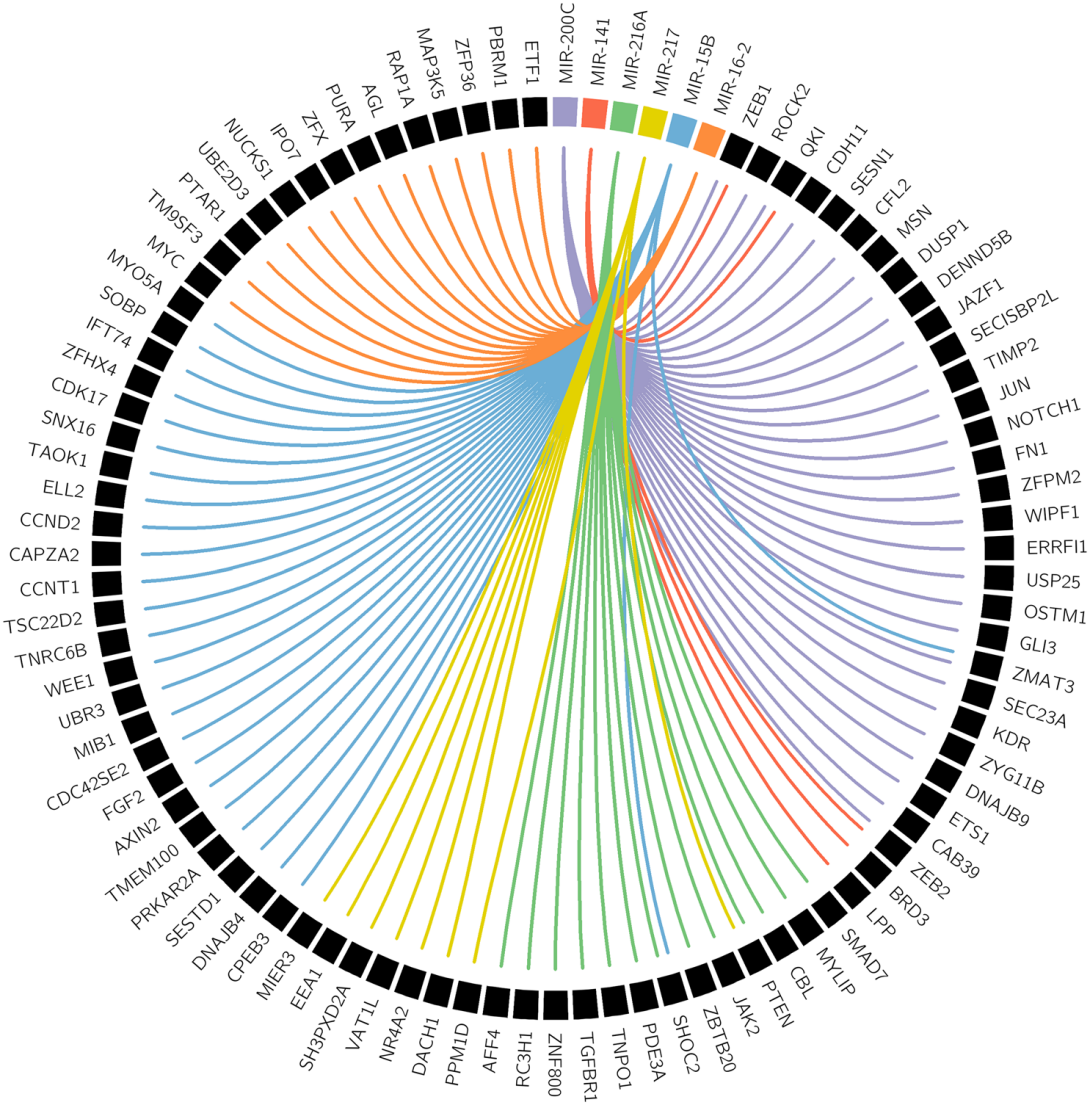
Interaction network of CNV driven miRNA clusters candidates and their mRNA targets. High confidence miRNA cluster targets were predicted with the help of five databases (miRTarBase, TargetScan, DIANA-TarBase, mirDIP and miRDB). The miRNA-mRNA regulatory networks were visualized by using Circos tool

The CNV driven, overexpressed three miRNA clusters target 180 genes. Promoter methylation (beta value) levels and the expression profile for all the target genes were given in Supplementary Tables S5 & S6. Among these, 64 genes were upregulated, and 27 were hyper-methylated. Hence, these 91 target genes were excluded from further analysis. The 89 genes downregulated in BCa targeted by clustered miRNAs from CNV gain regions are illustrated in Fig. 3. We assume that these genes were downregulated due to the upregulation of miRNA clusters. The expression of 89 target genes was cross-checked with BCa patient data available in GEO cohorts. Interestingly, 74 genes were significantly downregulated in BCa datasets (Supplementary Table S6). However, expression data for 15 genes were not found in the selected GEO datasets.

### PPI network construction and identification of hub genes

**Fig. 4:**
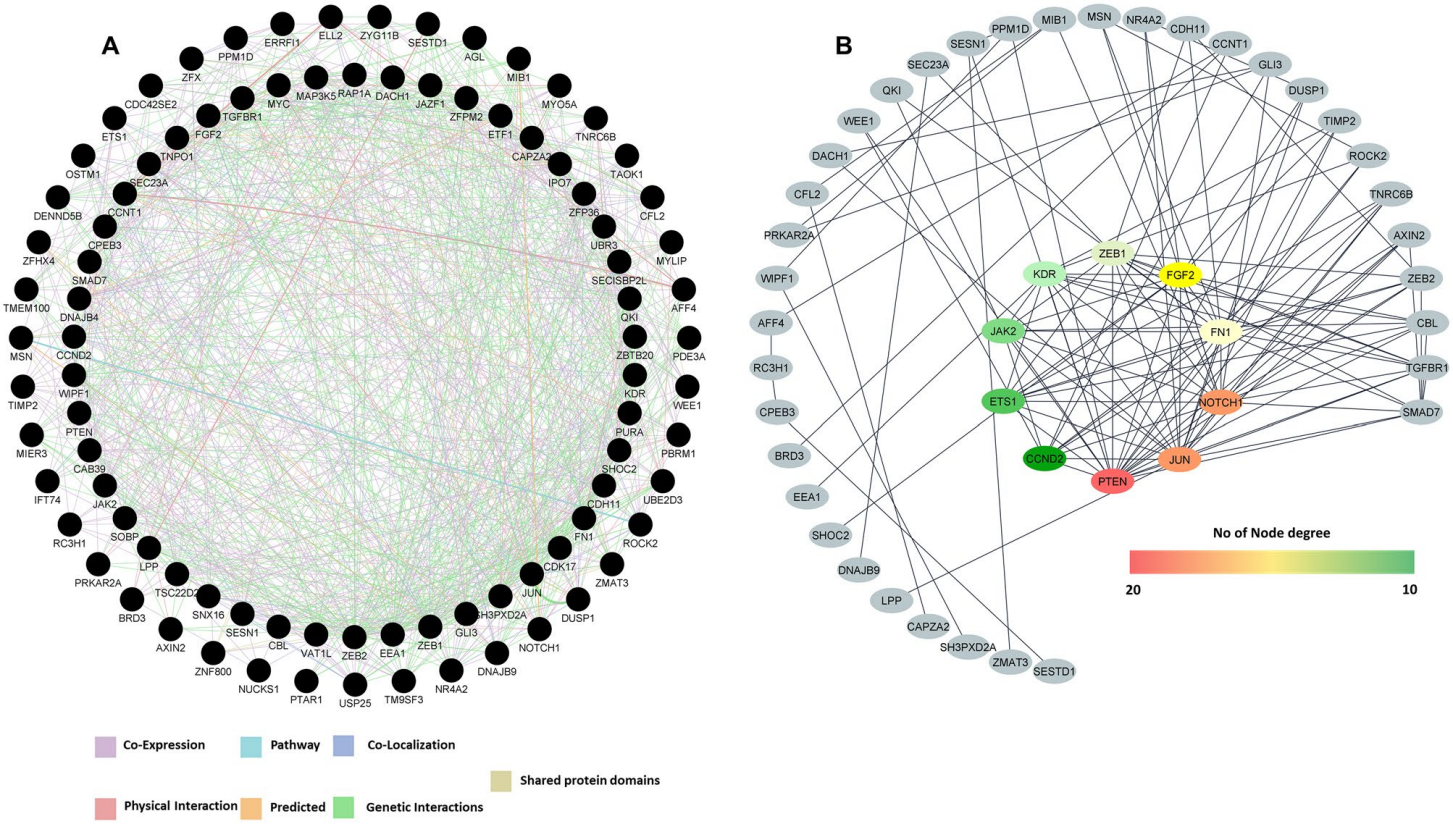
Protein–protein interaction network of CNV driven miRNA cluster targeted proteins. **a** Physical network of CNV driven miRNA cluster targeted proteins plotted with GeneMANIA (edge color: showing different interaction types; edge thickness: interaction strength; node: CNV driven miRNA cluster targets). **b** The interaction network of top 10 hub genes generated through CytoHubba plugin

We used the strength of existing network-based tools to study the protein–protein interaction and biological significance. The GeneMANIA resulted in genes and protein network shown in Fig. 4A highlights the functional association. Functionally associated interactions of 89 genes targeted by three miRNA clusters are given in Supplementary Table S7. According to STRING results, the PPI network of hsa-mir-200c/mir-141 cluster targets consists of 31 nodes and 36 edges, hsa-mir-216a/mir-217 cluster targets comprised 20 nodes and nine edges, and cluster hsa-mir-15b/mir-16-2 consists of 40 nodes and 23 edges. The connectivity degree of each node was calculated in these PPI networks and node degree > 10 was considered for hub genes (CCND2, ETS1, FGF2, FN1, JAK2, JUN, KDR, NOTCH1, PTEN, and ZEB1). All the hub genes showed a strong association with their node proteins (Fig. 4B). However, among these hub genes, PTEN showed the highest node degree (20), which is targeted by both the candidates of cluster hsa-mir-216a/mir-217. Among these hub genes, PTEN and ZEB1 are targeted by both the members of hsa-mir-216a/mir-217 and hsa-mir-200c/mir-141 clusters, respectively. Interestingly, mir-200c alone targets five of these hub genes (ETS1, FN1, JUN, KDR*,* and NOTCH1).

### GO term enrichment and KEGG pathway analysis

**Fig. 5:**
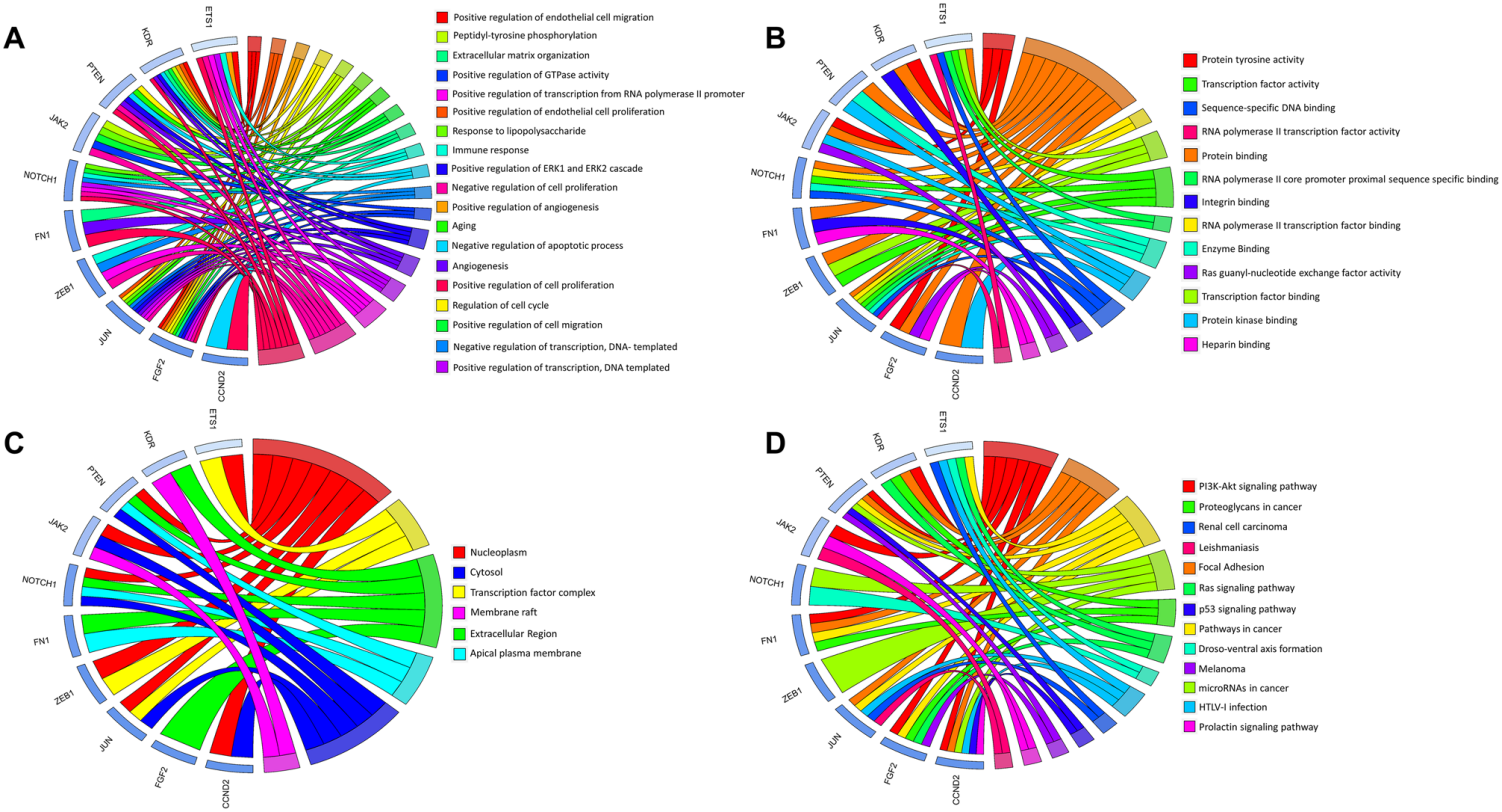
Functional enrichment analysis of the top 10 hub genes targeted by three miRNA clusters. **a** GO-BP (biological processes) enrichment, **b** GO-MF (Molecular functions) enrichment, **c** GO-CC (Cellular Component) enrichment, and **d** signaling pathway analysis was shown. The visual representation of functional enrichment analysis generated through GOPlot, a package of R

Gene ontology enrichment analysis of 89 genes targeted by three miRNA clusters is given in Supplementary Table S8. These genes are associated with 64 biological processes (BP), notably positive and negative regulation of cell proliferation, cell migration, signaling pathways, and positive regulation of gene expression. The enriched molecular functions (MF) include DNA binding, RNA binding, protein kinase activity, growth factor activity, metal ion binding, zinc ion binding, and translation repression. The enriched cellular component (CC) includes cytosol, cytoplasm, nucleoplasm, nucleus, centrosomes, membrane-bound vesicles, and cell projection. Furthermore, these genes were significantly associated with 14 pathways, including cancer signaling pathways such as p53, PI3K-Akt, MAPK, Wnt, and cAMP. The functional enrichment analysis of the top 10 hub genes belongs to the key cell signaling pathways such as p53, Ras, PI3K-Akt, prolactin signaling, and other carcinogenesis cascades. The hub genes associated with biological processes, molecular functions, cellular components, and enriched pathways are listed in Fig. 5.

### Tissue, molecular-subtype, and clinical traits specific expression analysis

**Fig. 6:**
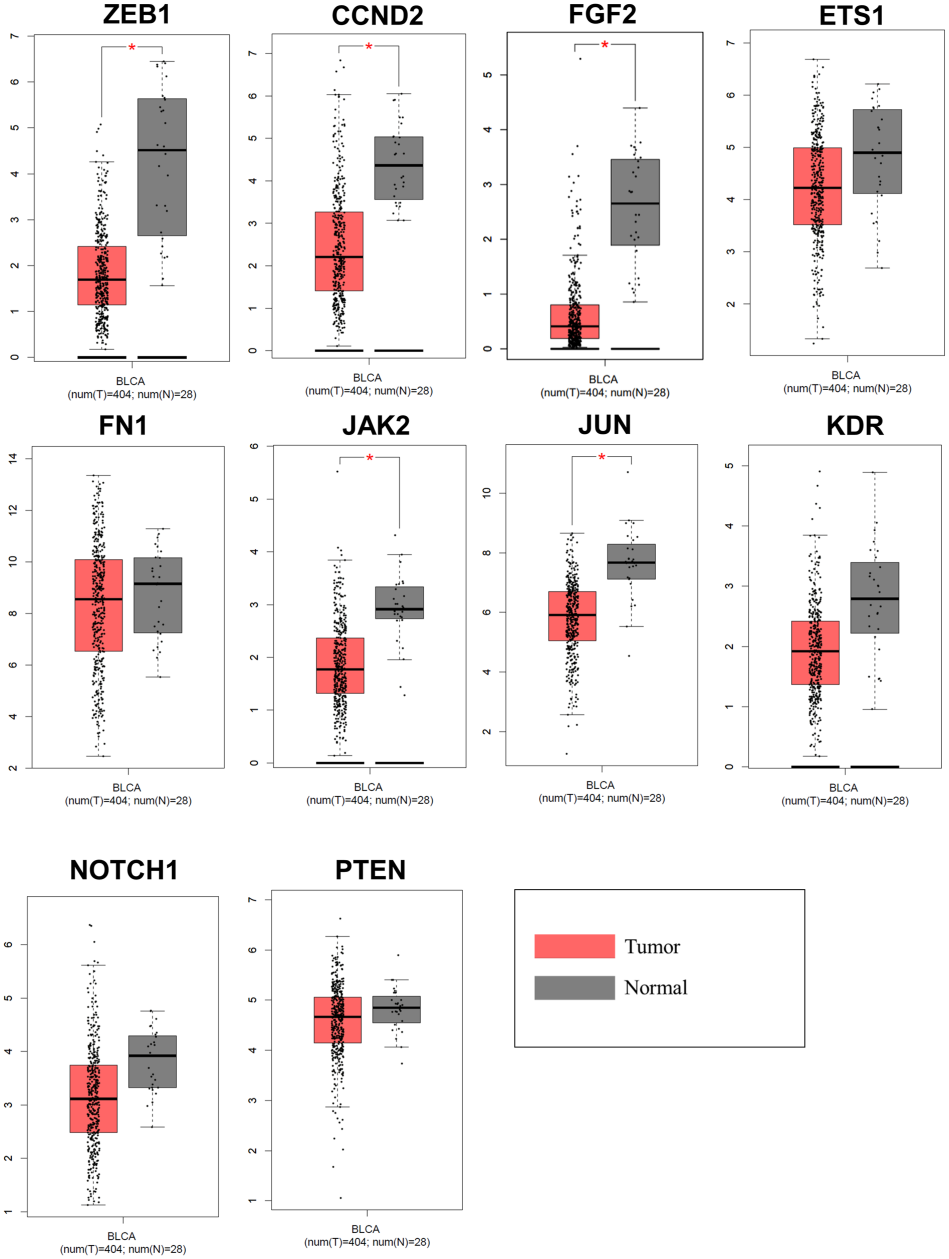
The differential expression of the top 10 hub genes in normal vs bladder cancer tissues (*p* value < 0.01). Expression data was procured from GEPIA, an online resource for gene expression profiling

Alongside this, the present study estimated the tissue-specific expression of hub genes in normal and bladder cancer tissues. The analysis also highlighted that ZEB1, CCND2, FGF2, JAK2, and JUN hub genes were significantly downregulated in BCa tissues (Fig. 6). Further, the examination of the CNV status of 10 hub genes showed that five genes reside on CNV regions (amplified: CCND2, KDR, and ZEB1; deleted: FN1 and PTEN). Hence, we hypothesize that the combined effect of CNV loss and miRNA binding might be responsible for the downregulation of FN1 and PTEN in BCa tissues. Subtype-specific expression patterns of clustered miRNA and hub genes were analyzed in BCa patients (Supplementary Fig. 1, 2). Proportionally, the total number of patients having downregulation (subtype-specific) of hub genes is more except FN1 (Basal Squamous & Luminal Infiltrated), KDR (Luminal, Luminal Infiltrated & Luminal Papillary), NOTCH1 (Basal Squamous). Expression profile analysis based on the smoking history of BCa patients vs. normal samples showed significant upregulation of mir-200c/141 and mir-15b/16-2 clusters in all the categories. We have observed expression trends of cluster targeted 10 hub genes are significantly downregulated in all categories of smoking history (Supplementary Fig. 3A). The BCa patient’s chemotherapy response trait information was procured for 236 samples from TCGA-GDC. Three miRNA clusters were upregulated in the responders and non-responders category except a miRNA mir-217 from mir-216a/217 cluster, which did not show a significant difference in expression compared with normal (Supplementary Fig. 3B). Interestingly, in the partial response group, NOTCH1 showed significant downregulation, but in the progressive and stable disease category, the expression was higher. Except for FN1, all other hub genes were downregulated in the therapy response category.

### Hub-genes and drug interactions

The integrated analysis of drug–gene interactions showed an association of seven hub genes with 20 potential FDA-approved drugs (Supplementary Table S9). Further, hub genes were analyzed for prioritizing the drugs and their clinical actionability for cancer therapy. Using this analysis, a total of 242 experimental drugs, 25 clinical trials, and one FDA-approved potential drug (methotrexate) were interacting with hub genes (Supplementary Table S10). These results could provide an opportunity for the repurposing of the drugs to treat BCa.

### Utility of the miRNA clusters for BCa staging

We have explored the expression pattern of CNV driven miRNA clusters in different stages of BCa (Supplementary Fig. 4). The stage-wise expression profile of both the candidates of hsa-mir-200c/mir-141 showed distinct signatures among BCa stages when compared to normal tissues (Supplementary Fig. 4A). The hsa-mir-15b/mir-16-2 candidates showed differences in the expression patterns in each stage of cancer progression (Supplementary Fig. 4C). Our analysis indicated that the candidate miRNA expression signature of hsa-mir-200c/mir-141 and hsa-mir-15b/mir-16-2 could differentiate healthy tissues from malignant phenotypes.

### CmiRClustFinder v1.0

**Fig. 7:**
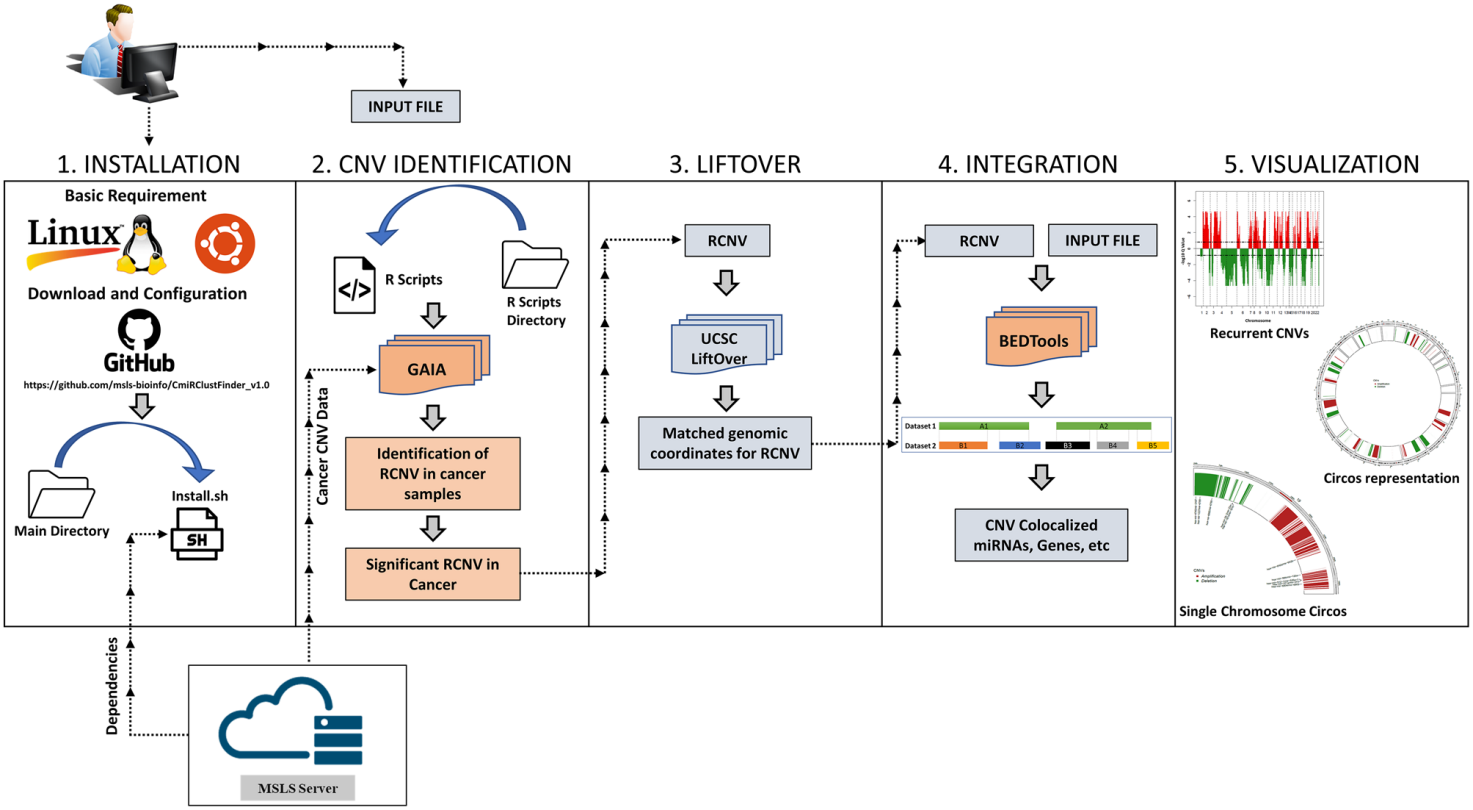
Five steps working process of the CmiRClustFinder v1.0 automated pipeline

Big data analytics through command line computation may be a daunting task to life science researchers. Hence, to overcome the computational challenges and reduce the complexity of multistep Commandline computing, we developed an automated pipeline called CmiRClustFinder v1.0 that computes the integrated data within five steps (Fig. 7). The installation script will download all the necessary resources and prepare the pipeline for use in the first step. In the second step, the GAIA package finds frequent aberrations in chromosomal regions among cancer patients’ datasets. In the third step, the LiftOver tool matches the genomic build for RCNVs and user-defined genetic elements. We have integrated BEDTools to find co-localization of significant RCNV and genomic elements in the fourth step. Lastly, the Circalize package generates a circos representation of the data. The source code for CmiRClustFinder v1.0 is publicly available at https://github.com/msls-bioinfo/CmiRClustFinder_v1.0. The manual for the pipeline execution is available in the portal.

## Discussion

Abnormal changes in miRNA expression may play a crucial role in the initiation, development, and progression of various cancers (Farazi et al. 2013). Since miRNA clusters contain multiple miRNAs encoding genes, their abnormal expression may collectively show a more severe impact on the cell signaling pathways than the individual miRNA. Recent reports have demonstrated clustered miRNAs cooperative and synergistic activity in various cancers (Cantini et al. 2019; Rui et al. 2020). Therefore, it is essential to explore the biological processes and cell signaling pathways affected due to dysregulated clustered miRNAs. Anauate et al. (2019) demonstrated copy number gain associated miRNA cluster of four miRNAs (miR-1207-3p, miR-1205, miR-1207-5p, and miR-1208) at 8q24 were upregulated ~ 50% of gastric tumors (Anauate et al. 2019). Our analysis identified 61 miRNA clusters that are localized within the CNV regions in BCa. To the best of our knowledge, this is the first comprehensive in silico study investigating the effect of recurrent CNV aberrations on miRNA cluster expression in BCa.

We identified prognostically significant three miRNA clusters, hsa-mir-200c/mir-141, hsa-mir-216a/mir-217, and hsa-mir-15b/mir-16-2 from BCa patients. Each cluster comprises of two miRNAs localized within the recurrent CNV gain regions were overexpressed. The aberrant expression of hsa-mir-200c/mir-141 has been reported in many human malignancies, where it participates in a variety of cellular processes such as epithelial-mesenchymal transition (EMT), proliferation, migration, invasion, and drug resistance (Senfter et al. 2016). Additionally, this cluster has been established as a potential diagnostic and prognostic biomarker for various carcinomas such as ovarian cancer (Gao et al. 2015), breast cancer (Choi et al. 2016), and lung adenocarcinoma (Tejero et al. 2014). Although the prognostic impact of hsa-mir-216a/mir-217 and hsa-mir-15b/mir-16-2 candidates’ dysregulation has been confirmed in several types of cancers (Aqeilan et al. 2010; Lovat et al. 2015; Azevedo-Pouly et al. 2017; Erener et al. 2021). However, there is no explicit evidence for these clusters can be used as a marker for BCa diagnosis.

Here, log-rank test-based Kaplan–Meier survival analysis showed hsa-mir-200c/mir-141 and hsa-mir-216a/mir-217 to be significantly correlated with the overall survival rate of BCa patients. The overexpression of mir-200c and mir-141 is strongly associated with a better prognosis in bladder cancer patients. Our findings are consistent with Mei et al. 2020, with additional evidence for CNV-induced overexpression of hsa-mir-200c/mir-141. ROC/AUROC analysis indicated high diagnostic accuracy for hsa-mir-200c/mir-141 and hsa-mir-15b/mir-16-2 cluster members. On the other hand, members of the hsa-mir-216a/mir-217 cluster exhibited no substantial sensitivity or specificity.

Further, members of these three miRNA clusters were subjected to target prediction, miRNA-mRNA network construction, PPI network analysis, pathway enrichment, and molecular function analysis to gain more insights. The study plotted a functional enrichment analysis of 89 genes that were targeted by three miRNA clusters to illustrate their association with different cancer signaling pathways. Network genes were filtered based on the number of nodes, and the top 10 higher degree node genes were denoted as hub genes (CCND2, ETS1, FGF2, FN1, JAK2, JUN, KDR, NOTCH1, PTEN, and ZEB1). The GO term and KEGG pathway enrichment analysis results indicated that these hub genes were related to the classical cancer signaling pathways. This includes previously defined p53, Ras, PI3K-Akt, and prolactin signaling. These pathways are closely correlated with proliferation, migration, invasion, and differentiation of the cancer cells (Sever and Brugge et al. 2015).

Following tissue-specific gene expression analysis from the GEPIA, GEO, and ULCAN datasets, the hub genes were shown to be considerably downregulated. The DNA promoter methylation analysis showed that these hub genes are not hyper-methylated in BCa. We observed that CCND2, KDR, and ZEB1 genes are residing on the CNV gain region while FN1 and PTEN are on CNV loss. The combined effect of CNV loss and miRNAs binding might be downregulating FN1 and PTEN in BCa tissues. The target identification studies showed that 109 non-clustered miRNAs are also targeting these 10 hub genes. However, the expression of these 109 non-clustered miRNAs was not statistically significant or less expressed in BCa tissues when compared to the six clustered miRNAs (Supplementary Table S11). Hence, the integrated analysis hypothesizes that miRNA clusters residing on CNV gain regions are potential regulators for bladder cancer.

Tobacco smoking is one of the most important risk factors for BCa with an attributable risk of approximately 50% (Cumberbatch et al. 2016). Tobacco-rich compounds such as aromatic amines and N-nitroso can induce DNA damage in the form of double-strand breaks, base modifications, or bulky adduct formation (Stern et al. 2009). Recent studies have suggested the possibility of tobacco smoking-induced oncogenic or antioncogenic gene expression by miRNA regulation in BCa (Cumberbatch et al. 2018; Navratilova et al. 2020). Hence, the analysis of the effect of recurrent genetic aberration in smoking and non-smoking groups of patients is essential. Considering this, BCa patients were grouped into five smoking categories (Lifelong Non-Smoker; Current Smoker; Current Reformed Smoker > 15 Years; Current Reformed Smoker ≤ 15 Years and Current Reformed Smoker; duration not specified) and expression analysis was performed. The members of mir-200c/mir-141 and mir-15b/mir-16-2 showed significant upregulation in each category. In comparison, no significant category-wise difference in mir-216a/mir-217 expression was identified. With the exception of FN1, the expression of other hub genes was significantly downregulated in all smoking groups.

The upregulated hsa-mir-200c/mir-141 targeted six hub genes: ETS1, FN1, JUN, KDR, NOTCH1, and ZEB1; all of which were downregulated in BCa. However, cancer stage-wise analysis showed decreased expression for hsa-mir-200c/mir-141 from stages 1 to 4 (Supplementary Fig. 4). In BCa, cluster hsa-mir-200c/mir-141 is frequently overexpressed and associated with early-stage (T1) bladder tumors (Han et al. 2011). Martínez-Fernández et al. (2015), suggested that increased expression of polycomb group protein BMI1 and EZH2 may contribute to the downregulation of hsa-mir-200c/mir-141 in high-grade stage BCa tumors through transcriptional repression. Downregulation of hsa-mir-200c/mir-141 leads to a subsequent upregulation of EMT promoting transcription factors (ZEB1 and ZEB2) and thus favors the invasive behavior of the tumor cells and cancer progression (Martínez-Fernández et al. 2015). Overexpression of hsa-mir-200c/mir-141 showed an improved survival rate in BCa patients with high specificity and sensitivity, suggesting it as a potential marker for BCa diagnosis.

The hsa-mir-15b/mir-16-2 cluster targeted CCND2 and FGF2 genes, were downregulated in BCa. The hyper-activation of CCND2 is generally considered as increased oncogenic activity in various tumors (Takano et al. 1999, 2000). Methylation of promoter region mediated silencing of CCND2 expression associated with a few cancer types progression is also reported (Evron et al. 2001; Wang et al. 2016). In the current analysis, identified CCND2 is unmethylated and resides on the CNV gain region in the tested bladder carcinoma datasets. Hence, we strongly assume that the downregulation of CCND2 might be due to the aberrant overexpression of hsa-mir-15b/mir-16-2 in malignant tumors.

The detailed functional annotation revealed that hsa-mir-216a/mir-217 targeted two hub genes (PTEN and JAK2), which act as tumor suppressors in numerous cancers (Qian et al. 2011; Lu et al. 2016). Studies have been reported that miRNAs can perform an oncogenic role by suppressing the function of PTEN in BCa (Feng et al. 2014), and the PTEN gene found to be associated with the CNV loss region. Therefore, the actual mechanism behind the downregulation of PTEN genes needs to be further investigated. Collectively, these findings strongly suggest that CNV driven overexpressed clustered miRNAs play an important role in regulating BCa signaling genes.

Resistance to treatment is one of the key problems associated with BCa patient survival. According to recent reports, miRNAs can also play a crucial role in the chemo-resistance mechanism in BCa (Senfter et al. 2016; Cai et al. 2019). Using drug–gene interaction analysis, we have identified 20 potential FDA-approved drugs interacting with miRNA clusters and their targeted genes in BCa. The PanDrugs drug–gene interactions analysis suggests that molecular alterations in the NOTCH1 gene are associated with high sensitivity to methotrexate (MTX). The MTX is commonly used as an anti-metabolite and chemotherapy for several cancer types (Hagner and Joerger 2020). High expression of Notch signaling genes has a vital role in many tumor cell’s resistance to methotrexate, while its downregulation increases drug sensitivity (Ma et al. 2013; Zhao et al. 2020). In the analysis of therapy responder and non-responder patients, we observed an upregulated expression of NOTCH1 in progressive and stable disease groups. Interestingly, in the disease progression group, the mir-200c/mir-141 was slightly downregulated. It suggests that the expression of mir-200c/mir-141 and NOTCH1 is inversely correlated. Also, in the partially responded group, NOTCH1 expression was downregulated. Hence, we assume that has-mir-200c/mir-141 cluster has a regulatory impact on NOTCH1, which may sensitize BCa cells to methotrexate.

In this in silico study, we have demonstrated the relationship between recurrent CNVs gain and their effect on the upregulation of miRNA clusters in BCa. The BCa stage-wise expression pattern of miRNA cluster candidates of has-mir-200c/mir-141 and hsa-mir-15b/mir-16-2 showed a distinct signature to differentiate healthy individuals from malignant phenotypes. These miRNA signatures can be used as potential prognostic BCa markers and cancer treatment. Collectively, the miRNA clusters upregulated by CNV gain may downregulate several cancer signaling genes and sensitize cancer cells to methotrexate.

Here, we report the first version of CmiRClustFinder, a computational pipeline that integrates multi-omics datasets such as CNV, miRNA, and gene expression to infer CNV driven clustered miRNAs from the TCGA cancer types. The CmiRClustFinder can be effectively utilized to identify novel miRNA biomarkers for various cancer types.

## Conclusion

Multi-omics approaches provided a large volume of genetic data for life science knowledge discovery. The study analyzed clustered miRNAs residing on CNV regions and developed an automated pipeline for the integrated data analysis. The integrated CNV-miRNA clusters data analysis identified 61 miRNA clusters (consisting of 153 miRNAs) residing on the CNV gain/loss region. The CNV driven, three prognostically significant miRNA clusters (hsa-mir-200c/141, hsa-mir-216a/mir-217, and hsa-mir-15b/mir-16-2) showed 2–fivefold increased expression in bladder cancer. Further, hsa-mir-200c/mir-141 and hsa-mir-15b/mir-16-2 clusters showed stage-wise difference in cancer progression. Interestingly, these clustered miRNAs targeted top 10 hub genes, the expression of which was downregulated in BCa tissues. Functional annotation indicates these hub genes have a key role in BCa and significantly impact patient survival and diagnosis. Hence, we hypothesize that these dysregulated clustered miRNAs can be used to screen bladder cancer progression as a potential diagnostic and prognostic indicator. Moreover, our integrated in silico results highlight a potential therapeutic application of clustered miRNA-based therapies for bladder cancer. The integrated analysis observed silencing of NOTCH1 by mir-200c/mir-141 improves methotrexate treatment and could benefit the BCa patient’s survival. The CmiRClustFinder pipeline can be used to identify novel clustered miRNAs to diagnose various cancer types.

## Supplementary Information

Below is the link to the electronic supplementary material.

Supplementary file1 (DOCX 1323 KB)

Supplementary file2 (XLSX 457 KB)

## Data Availability

Code for data analysis used in this study is provided on GitHub in the form of a pipeline. https://github.com/msls-bioinfo/CmiRClustFinder_v1.0.
