# Supplementary material for: Diagnostic and prognostic potential clustered miRNAs in bladder cancer
Source: 3 Biotech. 2022 Jul 13;12(8):173. doi: 10.1007/s13205-022-03225-z (PMC9279521; doi:10.1007/s13205-022-03225-z)
Supplement: Supplementary file 1 — Supplementary file1 (DOCX 1323 KB) [file 13205_2022_3225_MOESM1_ESM.docx]

**3Biotech**

**Diagnostic and Prognostic Potential Clustered miRNAs in Bladder Cancer**

**Supplementary information**

All Supplementary Tables are combined in a single Excel file.

Table titles and captions are listed below.

**Supplementary Table S1.** List of 4119 (1824 amplified, and 2295 deleted) significant CNV aberrations detected by GAIA analysis in BCa.

**Supplementary Table S2.** Details of 61 miRNA clusters residing on recurrent CNV regions (33 amplified and 28 deleted).

**Supplementary Table S3.** Details of nine miRNA clusters residing on CNV gain regions.

**Supplementary Table S4.** Differential expression values of nine miRNA cluster candidates in BCa.

**Supplementary Table S5.** The differential expression values for three CNV driven miRNA cluster candidates and its 74 target genes validated with GEO cohorts.

**Supplementary Table S6.** Details of expression values and DNA promoter methylation information of all identified target genes regulated by three miRNA clusters.

**Supplementary Table S7.** The functionally associated genes and their interaction results from GeneMANIA.

**Supplementary Table S8.** Gene ontology and pathway enrichment analysis result of 89 target genes generated by DAVID online platform.

**Supplementary Table S9.** Result of integrated analysis of drug-gene interactions.

**Supplementary Table S10.** The drug prioritization and inspection of their clinical actionability for therapy.

**Supplementary Table S11.** Details and expression of 109 non-clustered miRNAs targeting top 10 hub genes.

**Supplementary Table S12.** Details of up-regulated 49 oncogenes and downregulated 54 tumor suppressor genes identified by integrating datasets from TCGA-BLCA, Oncogene database (<http://ongene.bioinfo-minzhao.org/>) and TSGene database (<https://bioinfo.uth.edu/TSGene/>).

**Supplementary Figures**


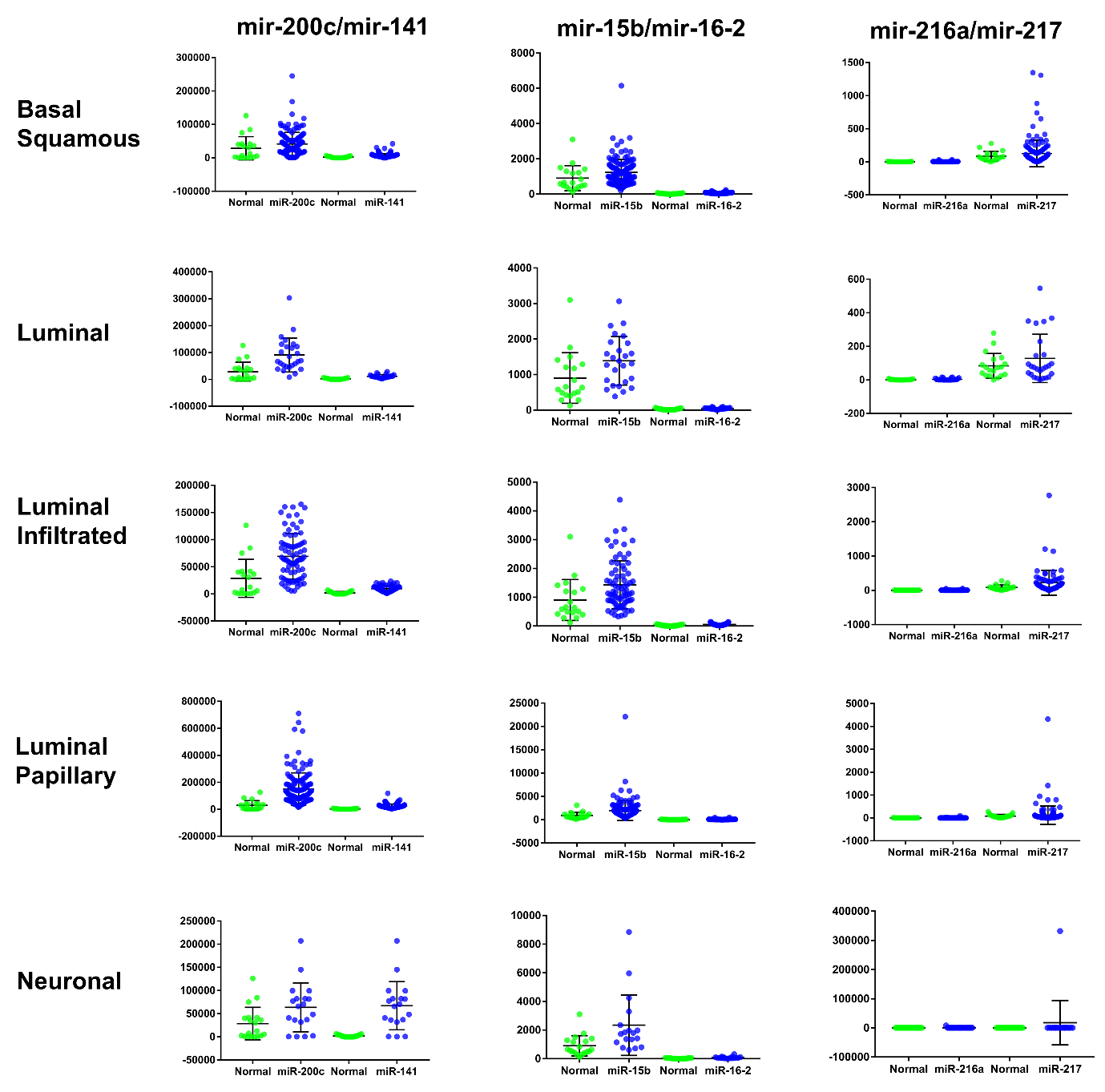


**Supplementary Fig. S1** Molecular subtype-specific expressions of clustered miRNAs.


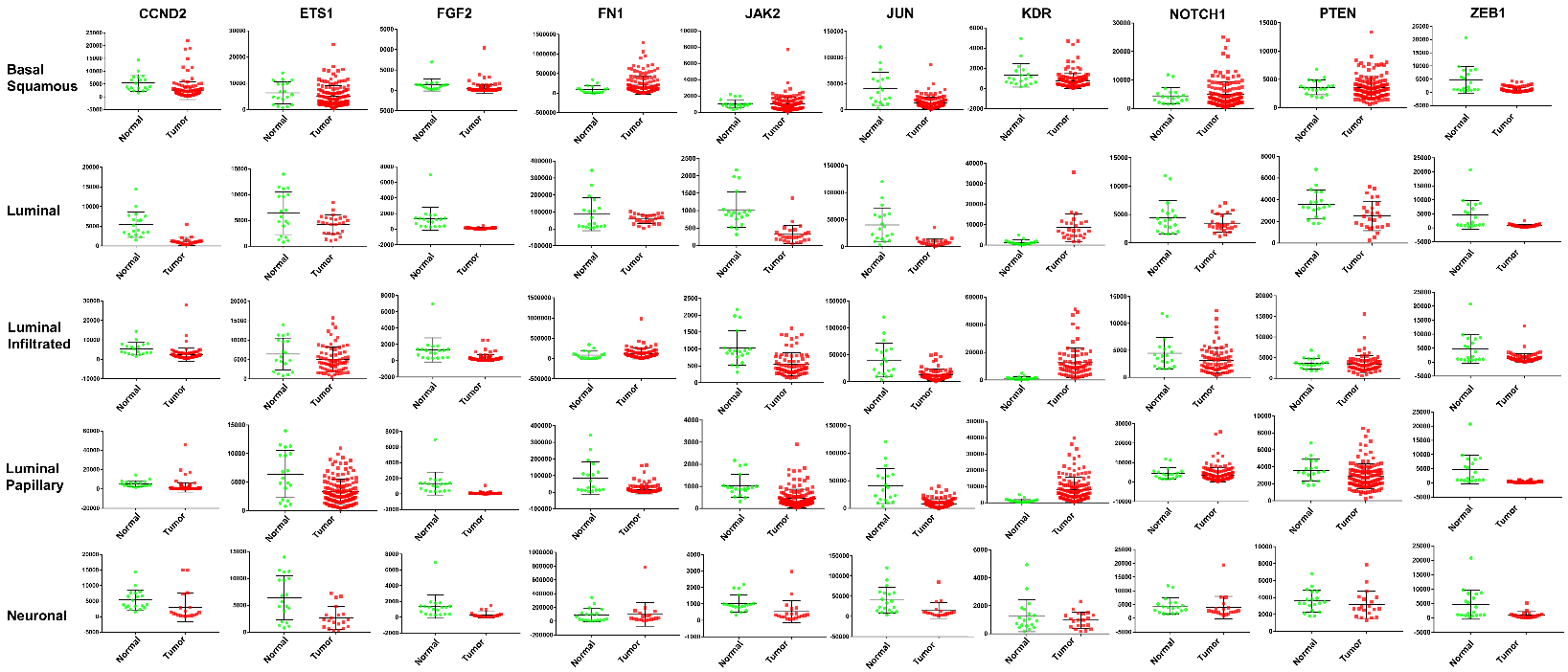


**Supplementary Fig. S2** Molecular subtype-specific expressions of top 10 hub genes.


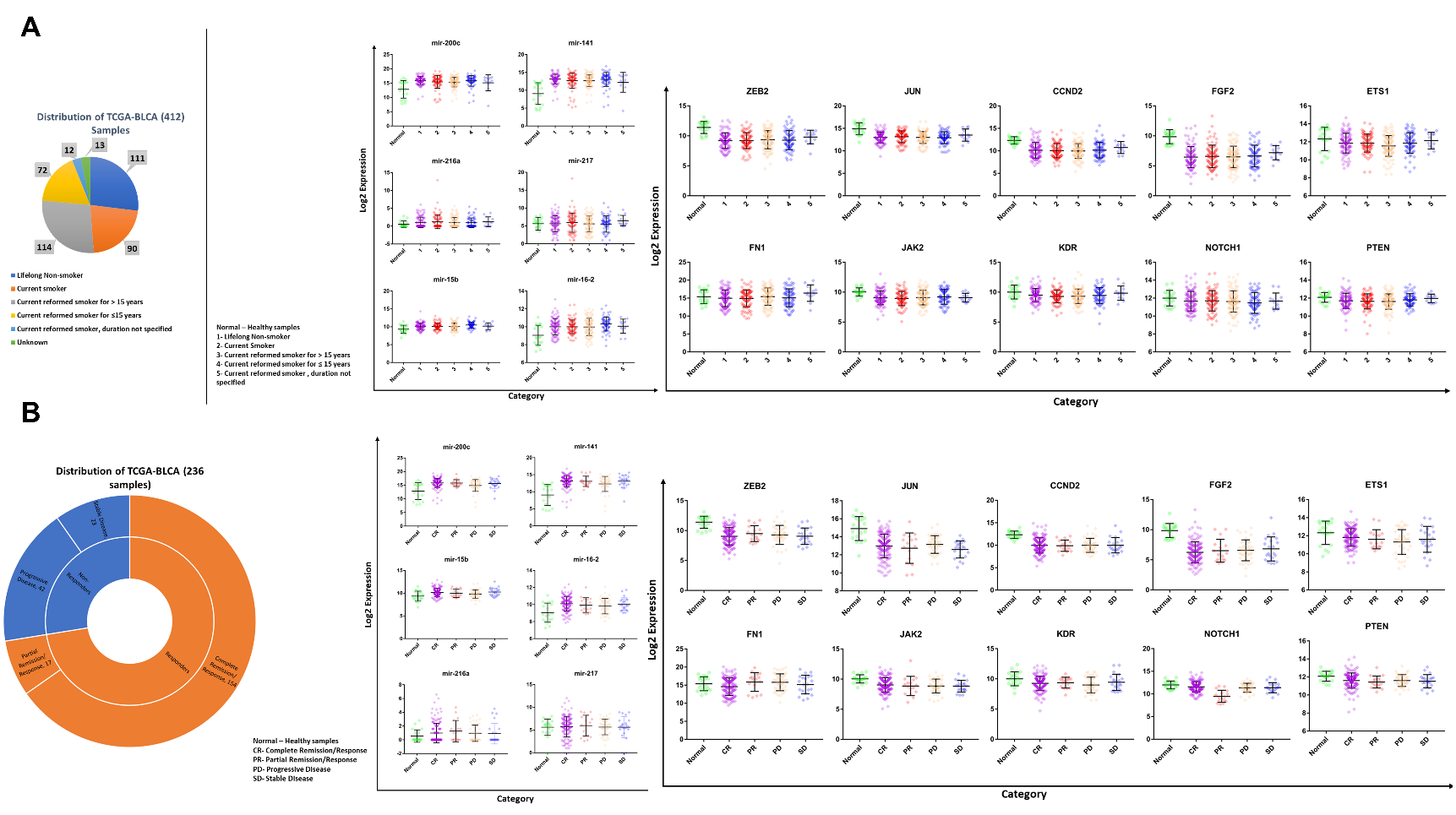


**Supplementary Fig. S3** Clustered miRNA and hub gene expression profiles based on smoking history and chemotherapy response patients group.


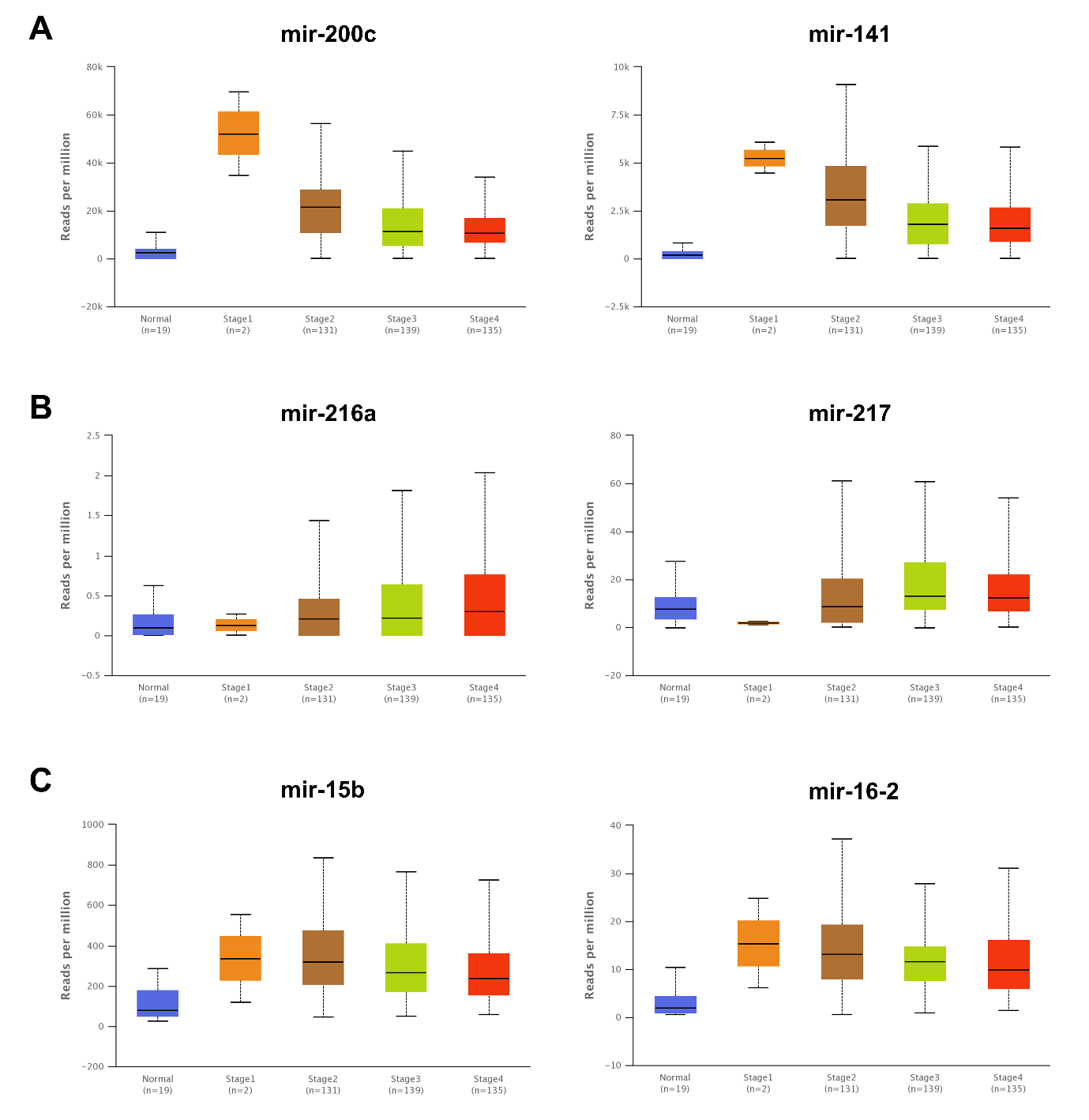


**Supplementary Fig. S4** The stage-wise expression pattern of miRNA cluster candidates in bladder cancer. a. has-mir-200c/mir-141, b. has-mir-216a/mir-217 and C. hsa-mir-15b/mir-16-2. The stage-wise expression obtained by UALCAN across all stages compared to normal tissues and expression was independent of the pathological stages (Stage 1: tumor is 7 cm across; Stage 2: tumor is larger than 7 cm across; Stage 3: tumor is growing and has spread to nearby; Stage 4: tumor is growing and has spread beyond the organs)
